# Utilizing Estimated Creatinine Excretion to Improve the Performance of Spot Urine Samples for the Determination of Proteinuria in Kidney Transplant Recipients

**DOI:** 10.1371/journal.pone.0166547

**Published:** 2016-12-02

**Authors:** Michael Ke Wang, Christine White, Ayub Akbari, Pierre Brown, Naser Hussain, Swapnil Hiremath, Greg Knoll

**Affiliations:** 1 Department of Medicine, McMaster University, Hamilton, Canada; 2 Division of Nephrology, Department of Medicine, Queens University, Kingston, Canada; 3 Division of Nephrology, Department of Medicine, University of Ottawa, Ottawa, Canada; 4 Kidney Research Centre, University of Ottawa, Ottawa, Canada; 5 Clinical Epidemiology Program, University of Ottawa, Ottawa, Canada; 6 Division of Nephrology, Department of Medicine, Mubarak AlKabeer Hospital, Kuwait City, Kuwait; The University of Manchester, UNITED KINGDOM

## Abstract

**Background:**

Agreement between spot and 24-hour urine protein measurements is poor in kidney transplant recipients. We investigated whether using formulae to estimate creatinine excretion rate (eCER), rather than assuming a standard creatinine excretion rate, would improve the estimation of proteinuria from spot urine samples in kidney transplant recipients.

**Methods:**

We measured 24 hour urine protein and albumin and spot albumin:creatinine (ACR) and spot protein:creatinine (PCR) in 181 Kidney transplant recipients.” We utilized 6 different published formulae (Fotheringham, CKD-EPI, Cockcroft-Gault, Walser, Goldwasser and Rule) to estimate eCER and from it calculated estimated albumin and protein excretion rate (eAER and ePER). Bias, precision and accuracy (within 15%, 30% and 50%) of ACR, PCR, eAER, ePER were compared to 24-hour urine protein and albumin.

**Results:**

ACR and PCR significantly underestimated 24-hour albumin and protein excretion (ACR Bias (IQR), -5.9 mg/day; p< 0.01; PCR Bias, (IQR), -35.2 mg/day; p<0.01). None of the formulae used to calculate eAER or ePER had a bias that was significantly different from the 24-hour collection (eAER and ePER bias: Fotheringham -0.3 and 7.2, CKD-EPI 0.3 and 13.5, Cockcroft-Gault -3.2 and -13.9, Walser -1.7 and 3.1, Goldwasser -1.3 and -0.5, Rule -0.6 and 4.2 mg/day respectively. The accuracy for ACR and PCR were lower (within 30% being 38% and 43% respectively) than the corresponding values estimated by utilizing eCER (for eAER 46% to 49% and ePER 46–54%).

**Conclusion:**

Utilizing estimated creatinine excretion to calculate eAER and ePER improves the estimation of 24-hour albuminuria/proteinuria with spot urine samples in kidney transplant recipients.

## Introduction

Albuminuria and proteinuria occur commonly in kidney transplant recipients. Both measurements have been associated with worsening kidney function, transplant loss, cardiovascular disease, and increased mortality[1–5]. The traditional reference standard for quantifying proteinuria and albuminuria has been the 24-hour urine collection. However, not only is the process of collection inconvenient for patients, but is also prone to both under- and over-collection errors[6]. In non-transplant clinical practice, the albumin-creatinine ratio (ACR) and protein-creatinine ratio (PCR) are commonly used as alternative methods to estimate albuminuria and proteinuria for reasons of cost and convenience. The KDIGO (Kidney Disease: Improving Global Outcomes) guidelines for the care of kidney transplant recipients, also suggest that ACR and PCR be used in kidney transplant recipients to quantify proteinuria[7]. In most instances, ACR and PCR are determined by measuring in a spot urine sample, albumin, protein and creatinine. The albumin concentration (for ACR) and protein concentration (for PCR) is divided by the measured urine creatinine to calculate ACR and PCR respectively.

Unfortunately, the absolute agreement between the ACR and PCR and the 24-hour urine collection is poor in both CKD and kidney transplant recipients [8, 9]. One possible explanation for the poor agreement is that ACR and PCR assume a creatinine (Cr) excretion rate of 1000 mg/day, an assumption that is not correct in many instances [10]. This may be even more relevant in the context of kidney transplantation where chronic steroid exposure could impact on muscle mass and turnover and hence affect Cr generation[11, 12]. Several formulae (Table 1) have been developed in non-transplant recipients to estimate Cr excretion rate (eCER) from patient demographics. We investigated whether using the estimated creatinine excretion rate (eCER) rather than a standard CER of 1000 mg/day (8.84 mmol/day), would improve the estimation of measured (24-hour) albumin excretion rate (mAER) and protein excretion rate (mPER) in kidney transplant recipients by spot urine samples.

**Table 1:** eCERFormulae.

	Formula	formula derived from, N (%, females)	Transplant patients
Fotheringham[9]	Male Black 1413.9 + (23.2 x age)—(0.3 x age^2^) Female Black 1148.6 + (15.6 x age)—(0.3 x age^2^) Male Nonblack 1307.3 + (23.1 x age)—(0.3 x age^2^) Female Nonblack 1051.3 + (5.3 x age)—(0.1 x age^2^)	1,693 (40)	None
CKD-EPI[13]	Male Black 879.89 + 12.51 x weight (Kg) -6.19 x age + 34.51 Female Black 879.89 + 12.51 x weight (Kg) -6.19 x age + 34.51–379.42 Male Nonblack 879.89 + 12.51 x weight (Kg) -6.19 x age Female Nonblack 879.89 + 12.51 x weight (Kg) -6.19 x age -379.42	1644 (41%)	None
Cockcroft-Gault[14]	Male [28—(0.2 x age)] x weight (Kg) Female [28—(0.2 x age)] x weight (Kg)x 0.85	249 (0%)	None
Walser[15]	Male (28.2—0.172 x age) x weight (Kg) Female (21.9—0.115 x age) x weight (Kg)	85 (37)	None
Goldwasser[16]	Black [(23.6—(age/8.3)+1.9] x weight (Kg) Nonblack 23.6—(age/8.3) x weight (Kg)	101(0%)	None
Rule[17]	Male [exp 7.26—(0.011 x (age—55) if age > 55)} x BSA/1.73 Female {[exp 7.26—0.26—(0.011 x (age—55) if age > 55)} x BSA/1.73	664 (51%)	None

## Subjects and Methods

### Study Population

This study was approved by the Ottawa Hospital Research Ethics Board. None of the transplant donors were from a vulnerable population and all donors or next of kin provided written informed consent that was freely given. We included adult kidney transplant recipients who had GFR measured and a 24-hour urine collection performed. A morning sample of urine was obtained to measure ACR and PCR on the same day the 24-hour collection was completed and returned to the laboratory. The patients were given detailed instruction on how to appropriately collect the 24 hour urine specimen. Inclusion criteria at the time of enrollment included: (1) stable transplant function (<0.4 mg/dL [35 μmol/L] difference in serum Cr between the 2 most recent values), and (2) at least 6 months post-transplantation. Those excluded: (1) were pregnant or breastfeeding, (2) had acute rejection within the preceding 3 months, (3) likely required kidney replacement therapy within the next 3 months, or (4) were likely to die within the next 3 months.

A total of 334 patients met the original study criteria and written consent was obtained from 261; a total of 54 patients withdrew from the study after consent was obtained but before investigations were conducted. Of the remaining 207 patients, 26 patients were further excluded for having missing data to calculate ACR or PCR, eAER or ePER or did not have a 24-hour urine collection. Thus, a total of 181 patients were included in the final analysis. Baseline characteristics including age, sex, race, cause of kidney failure, date of transplantation, donor type (living or deceased), and immunosuppression were recorded.

### Laboratory Assessment

Baseline urine and blood measurements were performed on the day of study entry. A 24-hour urine sample was collected for measurement of Cr, total protein and albumin. A morning urine sample was obtained to calculate spot ACR and PCR. All measurements were performed on a Beckman Coulter LX20 Pro Clinical System using Beckman Coulter reagents. Urine Cr was measured using non-isotope-dilution mass spectrometry standardized modified Jaffe Reaction. Coefficients of variation for urine Cr were 1.7% at 69 mg/dL (6.1 mmol/L) and 1.5% at 167 mg/dL (14.8 mmol/L). Urine protein was measured by pyrogallol red molybdate complex formation using a timed end point method. Coefficients of variation for urine protein were 3.6% at 0.02 g/dL (0.2 g/L) and 2% at 0.077 g/dL (0.77 g/L). Urine albumin was measured by an immunoturbidimetric method. Coefficients of variation for urine albumin were 4.1% at 2.7 mg/dL (27 mg/L) and 3.1% at 12 mg/dL (120 mg/L). Patients were weighed and had their height measured on the day the 24-hour urine collection was completed. Detailed standardized instructions were given to each patient for collection of 24 hour urine. GFR was measured by the plasma clearance of radiolabeled diethylenetriaminepentaacetic acid (99mTc-DTPA)[18, 19].

### Statistical Analysis

The primary aim of the analysis was to compare the performance of the eAER and ePER (calculated by multiplying ACR or PCR by eCER) to values obtained using the standard ACR and PCR (ACR or PCR mg/mg = grams per day of albumin or protein excretion)[10]. The 24-hour urine collection was considered the reference standard for the measurement of albumin (mAER) and protein (mPER). eCER was calculated using six different previously published formulae (Fotheringham[9], CKD-EPI[13], Cockcroft-Gault[14], Walser[15], Goldwasser[16], Rule[17]; Table 1). Median bias was calculated as value of albumin (or protein excretion) obtained by spot sample (eAER, ePER, ACR or PCR) minus the value obtained by 24-hour urine collection (reference standard). Percent median bias was calculated by dividing median bias of each observation by the corresponding mAER or mPER (bias/mAER or mPER)*100 and median of these values is reported as percent median bias. Accuracy of the eAER, ePER, ACR and PCR was determined by calculating the proportion of samples within 15% (P_15%_), 30% (P_30%_), and 50% (P_50%_) of the corresponding 24-hour urine value (i.e. the mAER or mPER)[9]. Exact 95% confidence intervals (CI) around accuracy measurements were calculated. Precision was assessed as the interquartile range of the median bias[8, 9]. Wilcoxon Rank-Sum Test was utilized to make the following comparisons: ACR versus mAER compared to eAER versus mAER and PCR versus mPER compared to ePER versus mPER. To account for multiple comparisons, we used a conservative Bonferroni correction factor. Since we compared seven values of estimated albumin excretion to the reference standard, we adjusted our overall alpha by 7 (overall α = 0.05/7 = 0.007), therefore a p-value of < 0.007 was considered statistically significant. To compare the accuracy, we utilized the McNemar’s test and compared accuracy of ACR and PCR to accuracy of eAER and ePER respectively. To account for multiple comparisons, we adjusted the overall alpha as above but for accuracy we had 6 comparisons (0.05/6 = 0.008) and as such for accuracy p < 0.008 was deemed statistically significant. Statistical analysis was performed using SPSS, version 21 (SPSS Inc) and MedCalc software version 16.2.0 (MedCalc Software, Ostend, Belgium; https://www.medcalc.org; 2016).

## Results

### Participant Characteristics

A total of 181 patients were included in the final analysis. Baseline characteristics of patients are presented in Table 2. The average age was 60.3 years. The majority had received deceased donor transplants (63%). The major causes of kidney disease were glomerulonephritis (26.5%), polycystic kidney disease (15.5%) and diabetes (13.3%) and most patients were on steroids (98.9%). The mean (SD) measured GFR was 59 (23) ml/min/1.73m^2^ and ranged from 11 to 128 ml/min/1.73m^2^. The median (IQR) mAER was 38 (14, 146)) mg/day and the median (IQR) mPER was 210 (70, 440) mg/day. Seventeen percent of patients had a mAER > 300 mg/day and 35% had a mPER > 300 mg/day. The median (IQR) spot PCR was 150.3 (86, 295) mg/g, and the median spot ACR (IQR) was 29.2 (12, 107) mg/g.

**Table 2:** Baseline Characteristics.

Age, years, Mean (SD)	60.3 (12.1)
Male, n (%)	62 (34.3)
Caucasian, n (%)	176 (97.2)
Weight, Kg, Mean (SD)	80.6 (17.8)
Height, cm, Mean (SD)	168 (9.9)
Living donor, n (%)	67 (37)
Time post-transplantation, years, Mean (SD)	7.3 (6.7)
***Cause of Kidney disease***	
Glomerulonephritis, n (%)	48 (26.5)
Polycystic kidney disease, n (%)	28 (15.5)
Diabetes, n (%)	24 (13.3)
Hypertension, n (%)	12 (6.6)
Other, n (%)	69 (38.1)
Measured GFR, ml/min/1.73m^2^, Mean (SD)	59.1 (23.2)
CKD stage 1, GFR ≥ 90 ml/min/1.73m^2^, n (%)	16 (8.8)
CKD stage 2, GFR ≥ 60 & < 90 ml/min/1.73m^2^, n (%)	68 (37.6)
CKD stage 3, GFR ≥ 30 & < 60 ml/min/1.73m^2^, n (%)	79 (43.6%)
CKD stage 4, GFR ≥ 15 & < 30 ml/min/1.73m^2^, n (%)	15 (8.3)
CKD stage 5, GFR < 15 ml/min/1.73m^2^, n (%)	3 (1.7)
***Medications***	
Steroids, n (%)	179 (98.9)
Cyclosporine, n (%)	95 (52.3)
Tacrolimus, n (%)	69 (38.1)
Sirolimus, n (%)	4 (2.2)
Mycophenolate mofetil, n (%)	121 (66.9)
Azathioprine, n (%)	31 (17.1)
24-hr albumin, mg/day, Median (IQR)	38 (14, 146)
24-hr protein, mg/day, Median (IQR)	210 (70, 440)
Albumin-creatinine ratio, Median (IQR)	29.2 (12, 107)
Protein-creatinine ratio, Median (IQR)	150.3 (86, 295)

GFR: Glomerular filtration rate. IQR: Interquartile range (Quartile 1, Quartile 3)

The results for albuminuria are shown in Tables 3 and 4. The ACR had the highest bias and significantly underestimated albumin excretion as compared to reference standard of mAER (median bias [IQR], -5.9 [32.2] mg/day; P < 0.01). In contrast, none of the eAER calculated by utilizing eCER from the six formulae was statistically different then reference standard (mAER) (Table 3). Numerically, the P_15%_, P_30%_ and P_50%_ for ACR were lower than the corresponding values estimated by the six eAERs. However, none of the six eAERs formulae used to estimate albumin excretion from the eCER was statistically different from the ACR (Table 4). Sub-group analysis by gender (Tables A-D in S1 Table) revealed that improvement in bias and accuracy was more notable in males.

**Table 3:** Bias and Precision of ACR and eAER compared to mAER.

N = 181	Median (IQR) Value [mg/day]	Median Bias [mg/24h]	% Median Bias	Precision [mg/24h]	P Value
mAER	38.0 (14, 146)	--	-	--	--
ACR	29.2 (12, 107)	-5.9	-25.5%	32.2	< 0.01*
**eAER by:**					
Fotheringham[9]	37.1 (14, 107)	-0.3	-3.2	29.7	0.91
CKD-EPI[13]	39.5 (14, 147)	0.3	1.6%	32.6	0.51
Cockcroft-Gault[14]	32.7 (13, 138)	-3.2	-15.4%	28.2	0.03
Walser[15]	35.0 (14, 141)	-1.7	-3.9%	31.8	0.92
Goldwasser[16]	35.2 (15, 143)	-1.3	-5.8%	30.9	0.46
Rule[17]	35.2 (14, 137)	-0.6	-4.0%	29.6	0.86

Median Bias: estimated value (either ACR or eAER)—measured value (mAER). % Median Bias: ((estimated value (either ACR or eAER)–measured value (mAER)/ measured value (mAER))*100. Precision: Interquartile range (IQR) of median bias. ACR: Albumin excretion rate calculated from albumin-creatinine ratio. eAER: Expected albumin excretion rate. mAER: Measured albumin excretion rate (24-hour urine albumin). P-value is for comparison between eAER or ACR and mAER.

*Indicates statistically significant result (P<0.007 considered statistically significant with Bonferroni correction for multiple comparisons; See Methods).

**Table 4:** Accuracy of ACR and eAER compared to mAER.

N = 181	P_15%_	P-value^α^	P_30%_	P-value^β^	P_50%_	P-value^μ^
ACR	18 (12, 24)	-	38 (31, 46)	-	64 (56, 71)	-
**eAER by:**						
Fotheringham[9]	24 (18, 30)	0.17	46 (38, 53)	0.13	64 (57, 71)	1.0
CKD-EPI[13]	24 (18, 31.0)	0.21	48 (41, 56)	0.05	70 (62, 76)	0.08
Cockcroft-Gault[14]	20 (14, 27)	0.66	48 (41, 56)	0.03	69 (62, 76)	0.10
Walser[15]	24 (18, 31.0)	0.19	49 (42, 57)	0.02	69 (62, 76)	0.11
Goldwasser[16]	25 (19, 32)	0.07	46 (39, 54)	0.07	68 (61, 75)	0.20
Rule[17]	25 (19, 32)	0.07	49 (42, 57)	0.02	67 (60, 74)	0.31

P_15%_, P_30%_, P_50%:_ Proportion of ACR or eAER within 15%, 30% and 50% of reference standard (measured 24-hour urine albumin) respectively. ^α^P, ^β^P, ^μ^P: P-value for comparison between accuracy of eAER vs accuracy of ACR for P_15%_, P_30%_ and P_50%_ respectively. ACR: Albumin excretion rate calculated from albumin-creatinine ratio. eAER: Expected albumin excretion rate. No result was statistically significant (P<0.008 considered statistically significant with Bonferroni correction for multiple comparisons; See Methods).

The results for proteinuria are shown in Tables 5 and 6. The PCR had the highest bias and significantly underestimated protein excretion as compared to reference standard of mPER (median bias [IQR], -35.2 [152] mg/day; P < 0.01). Numerically, the lowest bias was seen with the ePER calculated by Goldwasser formula (median bias [IQR], -0.5 [140] mg/day. None of the ePER calculated by utilizing eCER from the six formulae was statistically different from reference standard (mPER) (Table 5). Numerically, the P_15%_, P_30%_ and P_50%_ for PCR were all lower than the corresponding values estimated by the six formulae. In addition, the P_50%_ for the ePER determined using the eCER calculated from CKD-EPI, Walser and Rule formulae were all statistically greater than the PCR (Table 6). Sub-group analysis by gender (Tables E-I in S1 Table) revealed that improvement in bias and accuracy was more notable in males.

**Table 5:** Bias and Precision of PCR and ePER compared to mPER.

N = 181	Median (IQR) Value [mg/day]	Median Bias [mg/24h]	%Median Bias	Precision [mg/24h]	P Value
mPER	210 (70, 440)	-	-	-	-
PCR	150 (86, 295)	-35.2	-21.1	152	<0.01*
**ePER by:**					
Fotheringham[9]	19 (116, 397)	7.2	3.2	143	0.46
CKD-EPI[13]	19 (120, 395))	13.5	9.0	124	0.33
Cockcroft-Gault[14]	167 (98, 345)	-13.9	-9.0	143	0.009
Walser[15]	190(117, 392)	3.1	2.1	140	0.93
Goldwasser[16]	191(114, 391)	-0.5	-0.2	140	0.58
Rule[17]	189(106, 396)	4.2	3.5	121	0.93

Median Bias: estimated value (either PCR or ePER)—measured value (mPER). % Median Bias: ((estimated value (either PCR or ePER)–measured value (mPER)/ measured value (mPER))*100. Precision: Interquartile range (IQR) of median bias. PCR: Protein excretion rate calculated from protein-creatinine ratio. ePER: Expected protein excretion rate. mPER: Measured protein excretion rate (24-hour urine protein). P-value is for comparison between ePER or PCR and mPER.

*Indicates statistically significant result (P<0.007 considered statistically significant with Bonferroni correction for multiple comparisons; See Methods).

**Table 6:** Accuracy of PCR and ePER compared to mPER.

N = 181	P_15%_	P-value^α^	P_30%_	P-value^β^	P_50%_	P-value^μ^
PCR	22 (16, 29)		43(35, 50)		48(40, 55)	
**ePER by:**						
Fotheringham[9]	23(17, 30)	0.89	46(39, 54)	0.49	58(51, 65)	0.01
CKD-EPI[13]	25(19, 32)	0.43	54(46, 61)	0.03	60(53, 67)	<0.01*
Cockcroft-Gault[14]	25(19, 32)	0.50	48(41, 56)	0.21	54(46, 61)	0.14
Walser[15]	25(19, 32)	0.50	52(44, 59)	0.05	60(52, 67)	<0.01*
Goldwasser[16]	26(20, 33)	0.43	47(40, 55)	0.35	56(49, 64)	0.04
Rule[17]	27(20, 34)	0.34	53(45, 60)	0.04	73(67, 79)	<0.01*

P_15%_, P_30%_, P_50%:_ Proportion of PCR or ePER within 15%, 30% and 50% of reference standard (measured 24-hour urine protein) respectively. ^α^P, ^β^P, ^μ^P: P-value for comparison between accuracy of ePER vs accuracy of PCR for P_15%_, P_30%_ and P_50%_ respectively. PCR: Protein excretion rate calculated from protein-creatinine ratio. ePER: Expected protein excretion rate.

*Indicates statistically significant result (P<0.008 considered statistically significant with Bonferroni correction for multiple comparisons; See Methods).

## Discussion

In this study we found that the performance of spot urine samples for the measurement of albuminuria and proteinuria in transplant recipients can be improved by utilizing formulae to estimate CER, rather than assuming a constant CER. The median bias for the eAER using many of the formulae was quite low with several being less than 1 mg/day from the 24-hour urine collection and none was statistically different from the reference standard. In all cases, the accuracy of the formulae was numerically greater than the ACR or PCR but given adjustments for multiple testing only a few were statistically significant.

The studies that have examined the use of the eCER in proteinuria determination were performed in non-transplant recipients[9, 20–22]. Fotheringham showed improved bias and accuracy of the eAER calculated by the eCER using the Fotheringham formula[9] (bias 0.8 mg/24 h) as compared to the standard ACR (bias -3.8 mg/24 h). In the same study, the bias of the eAER calculated by eCER using CKD-EPI formula[13] was higher at 3.8 mg/24 h. This study did not assess any other formulae. Abdelmalek et al[21] also showed improved bias of CKD-EPI (median bias -1.0 mg/g), Fotheringham (-0.9 mg/g) and Walser (-0.8 mg/g) as compared to ACR (-2.3 mg/g). This study also revealed improved accuracy of the above three formulae as compared to ACR. More recently, Hong et al[20] published better correlation of eAER and ePER with 24 hour urine albumin and protein excretion. Finally, Bauer et al[22] revealed better association of eAER compared to ACR in predicting cardiovascular outcomes. In our study comprising only of transplant recipients, we found bias by eAER calculated by Fotheringham CER to be better than ACR and similar to eAER calculated by CKD-EPI formulae CER. Our results are slightly different from the non-transplant report probably because of the unique characteristics of our patients. The Fotheringham eCER formula does not include weight, whereas the CKD-EPI CER formula includes weight. Transplant recipients are chronically on steroids and may have less muscle mass and CER[11] and this may have contributed to the differing results.

Since accuracy takes into account both bias and precision, it is perhaps the best indicator of eAER and ePER performance in the clinical setting. We found the accuracy to be higher with eAER and ePER when compared to ACR and PCR. Given day to day variability of proteinuria (albuminuria), a P_30%_ greater than 80% seems likely to be a reasonable target for accuracy for clinical use. Despite the improvement of accuracy within 30% for both albuminuria (ACR, 38% versus eAER 46%-49%) and proteinuria (PCR 43% versus ePER 46%-54%), accuracy of spot urine samples remains modest at best in the kidney transplant population. Thus, a 24-hr urine collection should still be performed when protein or albumin excretion may alter major clinical decisions (e.g. considering kidney biopsy, immunosuppression change etc).

Given that the laboratories can automatically calculate eCER from Fotheringham formula, we suggest that the eCER calculated by Fotheringham formula be utilized to calculate eAER and ePER and that it should replace the use of ACR and PCR when routinely screening for albuminuria and proteinuria in the kidney transplant population.

Limitations to our study should be noted. First, only a small number of patients with protein and albumin excretion greater than 1000 mg/day were included, making it difficult to comment whether the improvements on bias and accuracy are applicable to patients with significant proteinuria. Second, we used formulae to estimate CER that have not been developed in the transplant population. Once available, such formulae may improve the accuracy and bias of eAER and ePER from spot samples in transplant recipients. Third, our study had a relatively small sample size and our findings require duplication in a larger cohort. Fourth, our study included predominantly Caucasians and the findings may not be generalizable to non-Caucasians who may have different muscle mass and CER[23]. Finally, although we provided detailed instructions on collection of 24 hour urine to our patients, unsupervised urine collections are prone to collection errors and improvement in precision (and achieving greater accuracy) may be limited by the inaccuracy of the reference standard.

In summary, the prediction of 24-hour protein and albumin excretion using spot samples can be improved by using formulae to estimate CER rather than assuming a constant CER. Unlike the standard ACR and PCR, the estimates of albuminuria/proteinuria using these formulae do not on average significantly underestimate protein (albumin) excretion in kidney transplant recipients. Overall accuracy, however, remains modest at best despite incremental improvements over the ACR and PCR. We suggest that laboratories consider reporting eAER and ePER calculated using the Fotheringham eCER formula as laboratories are able to automatically report these values (weight in not required in calculation). Validated formulae to estimate Cr excretion in transplant recipients are needed to determine if estimating albuminuria/proteinuria in this population can be further improved.

## Supporting Information

S1 Table Table A. Bias and Precision of ACR and eAER compared to mAER in Males
Table B. Bias and Precision of ACR and eAER compared to mAER in Females
Table C. Accuracy of ACR and eAER compared to mAER in Males
Table D. Accuracy of ACR and eAER compared to mAER in Females
Table E. Bias and Precision of PCR and ePER compared to mPER in Males
Table F. Bias and Precision of PCR and ePER compared to mPER in Females
Table G. Accuracy of PCR and ePER compared to mPER in Males
Table H. Accuracy of PCR and ePER compared to mPER in Females.
(DOCX)

S2 Table Table A. Bias and Precision of ACR and eAER compared to mAER in patients with albuminuria < 1 gram per day
Table B. Bias and Precision of ACR and eAER compared to mAER in patients with albuminuria > 1 gram per day
Table C. Accuracy of ACR and eAER compared to mAER in patients with albuminuria < 1 gram per day
Table D. Accuracy of ACR and eAER compared to mAER in patients with albuminuria > 1 gram per day
Table E. Bias and Precision of ACR and eAER compared to mAER in patients with mGFR > = 60 ml/min/1.73m^2^ (Stage 1 and 2 CKD)
Table F. Bias and Precision of ACR and eAER compared to mAER in patients with mGFR 30 to < 60 ml/min/1.73m^2^ (stage 3 CKD)
Table G. Bias and Precision of ACR and eAER compared to mAER in patients with mGFR < 30 ml/min/1.73m^2^ (Stage 4 and 5 CKD)
Table H. Accuracy of ACR and eAER compared to mAER in patients with mGFR > = 60 ml/min/1.73m^2^ (Stage 1 and 2 CKD)
Table I. Accuracy of ACR and eAER compared to mAER in patients with mGFR 30 to <60 ml/min/1.73m^2^ (Stage 3 CKD)
Table J. Accuracy of ACR and eAER compared to mAER in patients with mGFR < 30 ml/min/1.73m^2^ (Stage 4 and 5 CKD)
Table K. Bias and Precision of PCR and ePER compared to mPER with Proteinuria < 1 gram per day
Table L. Bias and Precision of PCR and ePER compared to mPER with Proteinuria >1 gram per day
Table M. Accuracy of PCR and ePER compared to mPER with Proteinuria < 1 gram per day
Table N. Accuracy of PCR and ePER compared to mPER with Proteinuria > 1 gram per day
Table O. Bias and Precision of PCR and ePER compared to mPER in patients with mGFR > = 60 ml/min/1.73m^2^ (Stage 1 and 2 CKD)
Table P. Bias and Precision of PCR and ePER compared to mPER in patients with mGFR 30 to <60 ml/min/1.73m^2^ (Stage 3 CKD)
Table Q. Bias and Precision of PCR and ePER compared to mPER in patients with mGFR < 30 ml/min/1.73m^2^ (Stage 4 and 5 CKD)
Table R. Accuracy of PCR and ePER compared to mPER in patients with mGFR > = 60 ml/min/1.73m^2^ (Stage 1 and 2 CKD)
Table S. Accuracy of PCR and ePER compared to mPER in patients with mGFR 30 to <60 ml/min/1.73m^2^ (Stage 3 CKD)
Table T. Accuracy of PCR and ePER compared to mPER in patients with mGFR < 30 ml/min/1.73m^2^ (Stage 4 and 5 CKD).
(DOCX)
