# Supplementary material for: Utilizing Estimated Creatinine Excretion to Improve the Performance of Spot Urine Samples for the Determination of Proteinuria in Kidney Transplant Recipients
Source: PLoS One. 2016 Dec 2;11(12):e0166547. doi: 10.1371/journal.pone.0166547 (PMC5135043; doi:10.1371/journal.pone.0166547)
Supplement: S1 Table — Table A. Bias and Precision of ACR and eAER compared to mAER in Males Table B. Bias and Precision of ACR and eAER compared to mAER in Females Table C. Accuracy of ACR and eAER compared to mAER in Males Table D. Accuracy of ACR and eAER compared to mAER in Females Table E. Bias and Precision of PCR and ePER compared to mPER in Males Table F. Bias and Precision of PCR and ePER compared to mPER in Females Table G. Accuracy of PCR and ePER compared to mPER in Males Table H. Accuracy of PCR and ePER compared to mPER in Females. (DOCX) [file pone.0166547.s001.docx]

**Supplementary 1 Table A: Bias and Precision of ACR and eAER compared to mAER in Males**

| **N= 119** | **Median (IQR) Value [mg/day]** | **Median Bias  [mg/24h]** | **% Median Bias** | **Precision**  **[mg/24h]** | **P Value** |
| --- | --- | --- | --- | --- | --- |
| mAER | 40 (16, 204) | -- | - | -- | -- |
| ACR | 30.9 (10-120) | -10.4 | -33.7% | 34.0 | **<0.01*** |
| **eAER by:** |  |  |  |  |  |
| Fotheringham([9](#_ENREF_9)) | 49.7 (17,193) | 0.6 | 2.6% | 36.4 | 0.32 |
| CKD-EPI([13](#_ENREF_13)) | 47.9 (15, 204) | 0.5 | 5.0% | 37.7 | 0.23 |
| Cockcroft-Gault([14](#_ENREF_14)) | 45.6 (13, 179) | -3.5 | -15.5% | 29.6 | 0.07 |
| Walser([15](#_ENREF_15)) | 49.3 (14, 199) | -1.8 | -3.9% | 35.0 | 0.94 |
| Goldwasser([16](#_ENREF_16)) | 44.0 (14, 187 | -2.6 | -10.9% | 33.0 | 0.21 |
| Rule([17](#_ENREF_17)) | 49.3 (14, 182) | -0.4 | -1.4% | 32.7 | 0.63 |

Median Bias: estimated value (either ACR or eAER) - measured value (mAER).

% Median Bias: ((estimated value (either ACR or eAER) – measured value (mAER)/ measured value (mAER))*100

Precision: Interquartile range (IQR) of median bias.

ACR: Albumin excretion rate calculated from albumin-creatinine ratio.

eAER: Expected albumin excretion rate.

mAER: Measured albumin excretion rate (24-hour urine albumin).

P-value is for comparison between eAER or ACR and mAER.

*Indicates statistically significant result (P<0.007 considered statistically significant with Bonferroni correction for multiple comparisons; See Methods).

**Supplementary 1 Table B: Bias and Precision of ACR and eAER compared to mAER in Females**

| **N= 62** | **Median (IQR) Value [mg/day]** | **Median Bias  [mg/24h]** | **% Median Bias** | **Precision**  **[mg/24h]** | **P Value** |
| --- | --- | --- | --- | --- | --- |
| mAER | 23 (13, 82) | -- | - | -- | -- |
| ACR | 22.5 (12, 88) | -1.1 | -9.8% | 21.6 | 0.30 |
| **eAER by:** |  |  |  |  |  |
| Fotheringham([9](#_ENREF_9)) | 23.1 (13, 82) | -1.6 | -9.2% | 22.3 | 0.22 |
| CKD-EPI([13](#_ENREF_13)) | 24.8 (13, 95) | -1.2 | -4.3% | 25.4 | 0.57 |
| Cockcroft-Gault([14](#_ENREF_14)) | 24.3 (13, 84) | -2.8 | -11.8% | 23.4 | 0.28 |
| Walser([15](#_ENREF_15)) | 26.3 (14, 98) | -0.8 | -3.7% | 24.7 | 0.88 |
| Goldwasser([16](#_ENREF_16)) | 28.7 (15, 114) | 0.4 | 5.0% | 18.3 | 0.53 |
| Rule([17](#_ENREF_17)) | 22.9 (13, 77) | -1.7 | -5.8% | 25.1 | 0.36 |

Median Bias: estimated value (either ACR or eAER) - measured value (mAER).

% Median Bias: ((estimated value (either ACR or eAER) – measured value (mAER)/ measured value (mAER))*100

Precision: Interquartile range (IQR) of median bias.

ACR: Albumin excretion rate calculated from albumin-creatinine ratio.

eAER: Expected albumin excretion rate.

mAER: Measured albumin excretion rate (24-hour urine albumin).

P-value is for comparison between eAER or ACR and mAER.

No result was statistically significant (P<0.007 considered statistically significant with Bonferroni correction for multiple comparisons; See Methods).

**Supplementary 1 Table C: Accuracy of ACR and eAER compared to mAER in Males**

| **N= 119** | **P_15%_** | **P-value^α^** | **P_30%_** | **P-value^β^** | **P_50%_** | **P-value^µ^** |
| --- | --- | --- | --- | --- | --- | --- |
| ACR | 16 (10, 24) | - | 36 (28, 46) | - | 65 (55, 73) | - |
| **eAER by:** |  |  |  |  |  |  |
| Fotheringham([9](#_ENREF_9)) | 26 (18, 35) | 0.08 | 47 (38, 56) | 0.03 | 66 (57, 75) | 0.73 |
| CKD-EPI([13](#_ENREF_13)) | 29 (21, 39) | 0.03 | 50 (40, 59) | 0.04 | 71 (62, 79) | 0.18 |
| Cockcroft-Gault([14](#_ENREF_14)) | 20 (13, 29) | 0.35 | 47 (38, 56) | 0.04 | 71 (63, 80) | 0.09 |
| Walser([15](#_ENREF_15)) | 27 (19, 36) | 0.04 | 52 (43, 61) | **<0.01*** | 70 (61, 78) | 0.24 |
| Goldwasser([16](#_ENREF_16)) | 26 (18, 35) | 0.05 | 52 (43, 61) | **<0.01*** | 73 (64, 81) | 0.03 |
| Rule([17](#_ENREF_17)) | 26 (18, 35) | 0.06 | 51 (42, 61) | 0.02 | 69 (60, 77) | 0.37 |

P_1 5%,_ P_30%,_ P_50%:_ Proportion of ACR or eAER within 15%, 30% and 50% of reference standard (measured 24-hour urine albumin) respectively.

^α^P, ^β^P, ^µ^P: P-value for comparison between accuracy of eAER vs accuracy of ACR for P_15%,_  P_30%_ and P_50%_ respectively.

ACR: Albumin excretion rate calculated from albumin-creatinine ratio.

eAER: Expected albumin excretion rate.

*Indicates statistically significant result (P<0.008 considered statistically significant with Bonferroni correction for multiple comparisons; See Methods).

**Supplementary 1 Table D: Accuracy of ACR and eAER compared to mAER in Females**

| **N= 62** | **P_15%_** | **P-value^α^** | **P_30%_** | **P-value^β^** | **P_50%_** | **P-value^µ^** |
| --- | --- | --- | --- | --- | --- | --- |
| ACR | 21 (12, 33) | - | 42 (30, 55) | - | 61 (48, 73) | - |
| **eAER by:** |  |  |  |  |  |  |
| Fotheringham([9](#_ENREF_9)) | 19 (10, 31) | 0.74 | 44 (31, 57) | 0.97 | 60 (47, 72) | 0.56 |
| CKD-EPI([13](#_ENREF_13)) | 13 (6, 24) | 0.13 | 45 (33, 58) | 0.59 | 68 (55, 79) | 0.10 |
| Cockcroft-Gault([14](#_ENREF_14)) | 19 (10, 31) | 0.81 | 50 (37, 63) | 0.23 | 65 (51, 76) | 0.48 |
| Walser([15](#_ENREF_15)) | 18 (9, 30) | 0.62 | 44 (31, 57) | 0.81 | 68 (55, 79) | 0.10 |
| Goldwasser([16](#_ENREF_16)) | 24 (14, 37) | 0.59 | 36 (24, 49) | 0.39 | 58 (45, 71) | 0.53 |
| Rule([17](#_ENREF_17)) | 23 (13, 35) | 0.76 | 45 (33, 58) | 0.53 | 65 (51, 76) | 0.32 |

P_1 5%,_ P_30%,_ P_50%:_ Proportion of ACR or eAER within 15%, 30% and 50% of reference standard (measured 24-hour urine albumin) respectively.

^α^P, ^β^P, ^µ^P: P-value for comparison between accuracy of eAER vs accuracy of ACR for P_15%,_  P_30%_ and P_50%_ respectively.

ACR: Albumin excretion rate calculated from albumin-creatinine ratio.

eAER: Expected albumin excretion rate.

No result was statistically significant (P<0.008 considered statistically significant with Bonferroni correction for multiple comparisons; See Methods).

**Supplementary 1 Table E:** **Bias and Precision of PCR and ePER compared to mPER in Males**

| **N=119** | **Median (IQR) Value [mg/day]** | **Median Bias  [mg/24h]** | **% Median Bias** | **Precision**  **[mg/24h]** | **P Value** |
| --- | --- | --- | --- | --- | --- |
| mPER | 240 (100, 480) | - |  | - | - |
| PCR | 151 (85, 326) | -62.5 | -28.5% | 153 | **<0.01*** |
| **ePER by:** |  |  |  |  |  |
| Fotheringham([9](#_ENREF_9)) | 238 (122, 528) | 16.4 | 9.9% | 162 | 0.20 |
| CKD-EPI([13](#_ENREF_13)) | 238 (132, 484) | 15.6 | 11.4% | 146 | 0.22 |
| Cockcroft-Gault([14](#_ENREF_14)) | 194 (109, 410) | -15.5 | -10.3% | 150 | 0.01 |
| Walser([15](#_ENREF_15)) | 216 (124, 452) | 3.1 | 2.1% | 151 | 0.92 |
| Goldwasser([16](#_ENREF_16)) | 202 (114, 406) | -9.7 | -5.9% | 153 | 0.08 |
| Rule([17](#_ENREF_17)) | 220 (119, 469) | 12.2 | 5.5% | 130 | 0.82 |

Median Bias: estimated value (either PCR or ePER) - measured value (mPER).

% Median Bias: ((estimated value (either PCR or ePER) – measured value (mPER)/ measured value (mPER))*100

Precision: Interquartile range (IQR) of median bias.

PCR: Protein excretion rate calculated from protein-creatinine ratio.

ePER: Expected protein excretion rate.

mPER: Measured protein excretion rate (24-hour urine protein).

P-value is for comparison between ePER or PCR and mPER.

*Indicates statistically significant result (P<0.007 considered statistically significant with Bonferroni correction for multiple comparisons; See Methods).

**Supplementary 1 Table F:** **Bias and Precision of PCR and ePER compared to mPER in Females**

| **N=62** | **Median (IQR) Value [mg/day]** | **Median Bias  [mg/24h]** | **% Median Bias** | **Precision**  **[mg/24h]** | **P Value** |
| --- | --- | --- | --- | --- | --- |
| mPER | 150 (70, 360) | - |  | - | - |
| PCR | 149 (88, 239) | -0.6 | -0.7% | 91 | 0.72 |
| **ePER by:** |  |  |  |  |  |
| Fotheringham([9](#_ENREF_9)) | 144 (87, 257) | -7.7 | -5.8% | 93 | 0.54 |
| CKD-EPI([13](#_ENREF_13)) | 161 (90, 250) | 0.4 | 0.5% | 98 | 0.89 |
| Cockcroft-Gault([14](#_ENREF_14)) | 148 (86, 281) | -9.9 | -8.7% | 107 | 0.38 |
| Walser([15](#_ENREF_15)) | 166 (94, 281) | 2.2 | 2.4% | 103 | 0.74 |
| Goldwasser([16](#_ENREF_16)) | 182 (103, 305) | 16.4 | 15.1% | 112 | 0.10 |
| Rule([17](#_ENREF_17)) | 148 (91, 238) | -5.5 | -5.7% | 113 | 0.58 |

Median Bias: estimated value (either PCR or ePER) - measured value (mPER).

% Median Bias: ((estimated value (either PCR or ePER) – measured value (mPER)/ measured value (mPER))*100

Precision: Interquartile range (IQR) of median bias.

PCR: Protein excretion rate calculated from protein-creatinine ratio.

ePER: Expected protein excretion rate.

mPER: Measured protein excretion rate (24-hour urine protein).

P-value is for comparison between ePER or PCR and mPER.

No result was statistically significant (P<0.007 considered statistically significant with Bonferroni correction for multiple comparisons; See Methods).

**Supplementary 1 Table G: Accuracy of PCR and ePER compared to mPER in Males**

| **N= 119** | **P_15%_** | **P-value^α^** | **P_30%_** | **P-value^β^** | **P_50%_** | **P-value^µ^** |
| --- | --- | --- | --- | --- | --- | --- |
| PCR | 18 (11, 26) | - | 43 (34, 52) | - | 47 (38, 56) | - |
| **ePER by:** |  |  |  |  |  |  |
| Fotheringham([9](#_ENREF_9)) | 21 (14, 29) | 0.55 | 45 (36, 55) | 0.72 | 61 (51, 69) | 0.03 |
| CKD-EPI([13](#_ENREF_13)) | 27 (19, 36) | 0.12 | 55 (45, 64) | 0.07 | 62 (54, 71) | 0.02 |
| Cockcroft-Gault([14](#_ENREF_14)) | 24 (17, 33) | 0.18 | 50 (40, 59) | 0.18 | 56 (46, 65) | 0.09 |
| Walser([15](#_ENREF_15)) | 28 (20, 37) | 0.05 | 52 (43, 61) | 0.12 | 62 (53, 71) | 0.01 |
| Goldwasser([16](#_ENREF_16)) | 28 (20, 37) | 0.05 | 50 (41, 60) | 0.14 | 56 (47, 65) | 0.07 |
| Rule([17](#_ENREF_17)) | 27 (19, 36) | 0.09 | 55 (45, 64) | 0.07 | 73 (64, 81) | **<0.01*** |

P_15%,_ P_30%,_ P_50%:_ Proportion of PCR or ePER within 15%, 30% and 50% of reference standard (measured 24-hour urine protein) respectively.

^α^P, ^β^P, ^µ^P: P-value for comparison between accuracy of ePER vs accuracy of PCR for P_15%,_  P_30%_ and P_50%_ respectively.

PCR: Protein excretion rate calculated from protein-creatinine ratio.

ePER: Expected protein excretion rate.

*Indicates statistically significant result (P<0.008 considered statistically significant with Bonferroni correction for multiple comparisons; See Methods).

**Supplementary 1 Table H: Accuracy of PCR and ePER compared to mPER in Females**

| **N= 62** | **P_15%_** | **P-value^α^** | **P_30%_** | **P-value^β^** | **P_50%_** | **P-value^µ^** |
| --- | --- | --- | --- | --- | --- | --- |
| PCR | 31 (20, 44) | - | 42 (30, 55) | - | 48 (36, 61) | - |
| **ePER by:** |  |  |  |  |  |  |
| Fotheringham([9](#_ENREF_9)) | 27 (17, 40) | 0.32 | 48 (36, 61) | 0.16 | 53 (40, 66) | 0.08 |
| CKD-EPI([13](#_ENREF_13)) | 23 (13, 35) | 0.20 | 52 (39, 65) | 0.11 | 57 (43, 69) | 0.10 |
| Cockcroft-Gault([14](#_ENREF_14)) | 26 (16, 39) | 0.51 | 45 (33, 58) | 0.62 | 50 (37, 63) | 0.78 |
| Walser([15](#_ENREF_15)) | 21 (12, 33) | 0.13 | 52 (39, 65) | 0.13 | 55 (42, 68) | 0.25 |
| Goldwasser([16](#_ENREF_16)) | 23 (13, 35) | 0.28 | 40 (28, 54) | 0.82 | 57 (43, 69) | 0.20 |
| Rule([17](#_ENREF_17)) | 26 (16, 39) | 0.37 | 48 (36, 61) | 0.21 | 73 (60, 83) | **<0.01*** |

P_15%,_ P_30%,_ P_50%:_ Proportion of PCR or ePER within 15%, 30% and 50% of reference standard (measured 24-hour urine protein) respectively.

^α^P, ^β^P, ^µ^P: P-value for comparison between accuracy of ePER vs accuracy of PCR for P_15%,_  P_30%_ and P_50%_ respectively.

PCR: Protein excretion rate calculated from protein-creatinine ratio.

ePER: Expected protein excretion rate.

*Indicates statistically significant result (P<0.008 considered statistically significant with Bonferroni correction for multiple comparisons; See Methods).
