# Supplementary material for: Utilizing Estimated Creatinine Excretion to Improve the Performance of Spot Urine Samples for the Determination of Proteinuria in Kidney Transplant Recipients
Source: PLoS One. 2016 Dec 2;11(12):e0166547. doi: 10.1371/journal.pone.0166547 (PMC5135043; doi:10.1371/journal.pone.0166547)
Supplement: S2 Table — Table A. Bias and Precision of ACR and eAER compared to mAER in patients with albuminuria < 1 gram per day Table B. Bias and Precision of ACR and eAER compared to mAER in patients with albuminuria > 1 gram per day Table C. Accuracy of ACR and eAER compared to mAER in patients with albuminuria < 1 gram per day Table D. Accuracy of ACR and eAER compared to mAER in patients with albuminuria > 1 gram per day Table E. Bias and Precision of ACR and eAER compared to mAER in patients with mGFR > = 60 ml/min/1.73m2 (Stage 1 and 2 CKD) Table F. Bias and Precision of ACR and eAER compared to mAER in patients with mGFR 30 to < 60 ml/min/1.73m2 (stage 3 CKD) Table G. Bias and Precision of ACR and eAER compared to mAER in patients with mGFR < 30 ml/min/1.73m2 (Stage 4 and 5 CKD) Table H. Accuracy of ACR and eAER compared to mAER in patients with mGFR > = 60 ml/min/1.73m2 (Stage 1 and 2 CKD) Table I. Accuracy of ACR and eAER compared to mAER in patients with mGFR 30 to <60 ml/min/1.73m2 (Stage 3 CKD) Table J. Accuracy of ACR and eAER compared to mAER in patients with mGFR < 30 ml/min/1.73m2 (Stage 4 and 5 CKD) Table K. Bias and Precision of PCR and ePER compared to mPER with Proteinuria < 1 gram per day Table L. Bias and Precision of PCR and ePER compared to mPER with Proteinuria >1 gram per day Table M. Accuracy of PCR and ePER compared to mPER with Proteinuria < 1 gram per day Table N. Accuracy of PCR and ePER compared to mPER with Proteinuria > 1 gram per day Table O. Bias and Precision of PCR and ePER compared to mPER in patients with mGFR > = 60 ml/min/1.73m2 (Stage 1 and 2 CKD) Table P. Bias and Precision of PCR and ePER compared to mPER in patients with mGFR 30 to <60 ml/min/1.73m2 (Stage 3 CKD) Table Q. Bias and Precision of PCR and ePER compared to mPER in patients with mGFR < 30 ml/min/1.73m2 (Stage 4 and 5 CKD) Table R. Accuracy of PCR and ePER compared to mPER in patients with mGFR > = 60 ml/min/1.73m2 (Stage 1 and 2 CKD) Table S. Accuracy of PCR and ePER compared to m [file pone.0166547.s002.docx]

| **N= 169** | **Median (IQR) Value [mg/day]** | **Median Bias  [mg/24h]** | **% Median Bias** | **Precision**  **[mg/24h]** | **P Value** |
| --- | --- | --- | --- | --- | --- |
| mAER | 34 (14, 95) | -- | - | -- | -- |
| ACR | 26.5 (11, 84) | -5.2 | -24.9% | 23.5 | **<0.01*** |
| **eAER by:** |  |  |  |  |  |
| Fotheringham([9](#_ENREF_9)) | 34 (13, 108) | -0.2 | -0.5% | 27.7 | 0.54 |
| CKD-EPI([13](#_ENREF_13)) | 32 (13, 109) | -0.5 | 3.3% | 26.8 | 0.25 |
| Cockcroft-Gault([14](#_ENREF_14)) | 29.1 (13, 98) | -2.9 | -12.6% | 26.7 | 0.1 |
| Walser([15](#_ENREF_15)) | 31.5 (14, 110) | -0.8 | -3.5% | 25.8 | 0.70 |
| Goldwasser([16](#_ENREF_16)) | 32 (15, 112) | -1 | -4.8% | 27.3 | 0.83 |
| Rule([17](#_ENREF_17)) | 31.7 (13, 106) | -0.4 | -1.4% | 25.6 | 0.74 |

**Supplementary 2 Table A: Bias and Precision of ACR and eAER compared to mAER in patients with albuminuria < 1 gram per day**

Median Bias: estimated value (either ACR or eAER) - measured value (mAER).

% Median Bias: ((estimated value (either ACR or eAER) – measured value (mAER)/ measured value (mAER))*100

Precision: Interquartile range (IQR) of median bias.

ACR: Albumin excretion rate calculated from albumin-creatinine ratio.

eAER: Expected albumin excretion rate.

mAER: Measured albumin excretion rate (24-hour urine albumin).

P-value is for comparison between eAER or ACR and mAER.

*Indicates statistically significant result (P<0.007 considered statistically significant with Bonferroni correction for multiple comparisons; See Methods).

**Supplementary 2 Table B: Bias and Precision of ACR and eAER compared to mAER in patients with albuminuria > 1 gram per day**

| **N= 12** | **Median (IQR) Value [mg/day]** | **Median Bias  [mg/24h]** | **% Median Bias** | **Precision**  **[mg/24h]** | **P Value** |
| --- | --- | --- | --- | --- | --- |
| mAER | 1576 (1317, 2378) | -- | - | -- | -- |
| ACR | 1127 (629, 1967) | -486 | -32% | 641 | **<0.01*** |
| **eAER by:** |  |  |  |  |  |
| Fotheringham([9](#_ENREF_9)) | 1226 (915, 2559) | -209 | -13.3% | 1074 | 0.52 |
| CKD-EPI([13](#_ENREF_13)) | 1321 (849, 2521) | -103 | -8.3% | 967 | 0.62 |
| Cockcroft-Gault([14](#_ENREF_14)) | 1196 (673, 1856) | -354 | -27.6% | 888 | 0.08 |
| Walser([15](#_ENREF_15)) | 1345 (783, 2161) | -225 | -15.8% | 905 | 0.3 |
| Goldwasser([16](#_ENREF_16)) | 1280 (805, 2041) | -294 | -20.6% | 817 | 0.2 |
| Rule([17](#_ENREF_17)) | 1264 (862, 2372) | -314 | -18.5% | 856 | 0.42 |

Median Bias: estimated value (either ACR or eAER) - measured value (mAER).

% Median Bias: ((estimated value (either ACR or eAER) – measured value (mAER)/ measured value (mAER))*100

Precision: Interquartile range (IQR) of median bias.

ACR: Albumin excretion rate calculated from albumin-creatinine ratio.

eAER: Expected albumin excretion rate.

mAER: Measured albumin excretion rate (24-hour urine albumin).

P-value is for comparison between eAER or ACR and mAER.

*Indicates statistically significant result (P<0.007 considered statistically significant with Bonferroni correction for multiple comparisons; See Methods).

**Supplementary 2 Table C: Accuracy of ACR and eAER compared to mAER in patients with albuminuria < 1 gram per day**

| **N= 169** | **P_15%_** | **P-value^α^** | **P_30%_** | **P-value^β^** | **P_50%_** | **P-value^µ^** |
| --- | --- | --- | --- | --- | --- | --- |
| ACR | 17 (11, 23) | - | 38 (31, 46) | - | 63 (55, 70) | - |
| **eAER by:** |  |  |  |  |  |  |
| Fotheringham([9](#_ENREF_9)) | 24 (18, 31) | 0.08 | 46 (38, 53) | 0.15 | 63 (56, 71) | 0.91 |
| CKD-EPI([13](#_ENREF_13)) | 24 (18, 31) | 0.1 | 48 (40, 56) | 0.06 | 69 (62, 76) | 0.21 |
| Cockcroft-Gault([14](#_ENREF_14)) | 19 (13, 26) | 0.57 | 49 (41, 56) | 0.05 | 68 (61, 75) | 0.3 |
| Walser([15](#_ENREF_15)) | 24 (18, 31) | 0.1 | 49 (41, 56) | 0.05 | 69 (61, 76) | 0.25 |
| Goldwasser([16](#_ENREF_16)) | 25 (19, 33) | 0.05 | 47 (39, 55) | 0.1 | 67 (59, 74) | 0.43 |
| Rule([17](#_ENREF_17)) | 25 (19, 32) | 0.06 | 49 (41, 56) | 0.05 | 67 (59, 74) | 0.43 |

P_1 5%,_ P_30%,_ P_50%:_ Proportion of ACR or eAER within 15%, 30% and 50% of reference standard (measured 24-hour urine albumin) respectively.

^α^P, ^β^P, ^µ^P: P-value for comparison between accuracy of eAER vs accuracy of ACR for P_15%,_  P_30%_ and P_50%_ respectively.

ACR: Albumin excretion rate calculated from albumin-creatinine ratio.

eAER: Expected albumin excretion rate.

No result was statistically significant (P<0.008 considered statistically significant with Bonferroni correction for multiple comparisons; See Methods).

**Supplementary 2 Table D: Accuracy of ACR and eAER compared to mAER in patients with albuminuria > 1 gram per day**

| **N= 12** | **P_15%_** | **P-value^α^** | **P_30%_** | **P-value^β^** | **P_50%_** | **P-value^µ^** |
| --- | --- | --- | --- | --- | --- | --- |
| ACR | 33 (10, 65) | - | 42 (15, 72) | - | 75 (43, 95) | - |
| **eAER by:** |  |  |  |  |  |  |
| Fotheringham([9](#_ENREF_9)) | 17 (2, 48) | 0.36 | 50 (21, 79) | 0.69 | 75 (43, 95) | 1.0 |
| CKD-EPI([13](#_ENREF_13)) | 25 (6, 57) | 0.66 | 50 (21, 79) | 0.69 | 75 (43, 95) | 1.0 |
| Cockcroft-Gault([14](#_ENREF_14)) | 33 (10, 65) | 1.0 | 42 (15, 72) | 1 | 83 (52, 98) | 0.62 |
| Walser([15](#_ENREF_15)) | 25 (6, 57) | 0.66 | 58 (28, 85) | 0.42 | 75 (43, 95) | 1.0 |
| Goldwasser([16](#_ENREF_16)) | 25 (6, 57) | 0.66 | 42 (15, 72) | 1 | 83 (52, 98) | 0.62 |
| Rule([17](#_ENREF_17)) | 25 (6, 57) | 0.66 | 58 (28, 85) | 0.42 | 75 (43, 95) | 1.0 |

P_1 5%,_ P_30%,_ P_50%:_ Proportion of ACR or eAER within 15%, 30% and 50% of reference standard (measured 24-hour urine albumin) respectively.

^α^P, ^β^P, ^µ^P: P-value for comparison between accuracy of eAER vs accuracy of ACR for P_15%,_  P_30%_ and P_50%_ respectively.

ACR: Albumin excretion rate calculated from albumin-creatinine ratio.

eAER: Expected albumin excretion rate.

No result was statistically significant (P<0.008 considered statistically significant with Bonferroni correction for multiple comparisons; See Methods).

| **N= 84** | **Median (IQR) Value [mg/day]** | **Median Bias  [mg/24h]** | **% Median Bias** | **Precision**  **[mg/24h]** | **P Value** |
| --- | --- | --- | --- | --- | --- |
| mAER | 26.5 (14, 269 | -- | - | -- | -- |
| ACR | 22.1 (9, 70) | -5.2 | -23.5% | 24.9 | **<0.01*** |
| **eAER by:** |  |  |  |  |  |
| Fotheringham([9](#_ENREF_9)) | 26.3 (13, 88) | -0.5 | -3.5% | 27.4 | 0.77 |
| CKD-EPI([13](#_ENREF_13)) | 25.3 (13, 96) | 0.4 | 4.3% | 29.1 | 0.6 |
| Cockcroft-Gault([14](#_ENREF_14)) | 22.6 (12, 70) | -1.7 | -10.4% | 23.4 | 0.38 |
| Walser([15](#_ENREF_15)) | 25.6 (13, 81) | 0.15 | 0.7% | 24.6 | 0.7 |
| Goldwasser([16](#_ENREF_16)) | 26.6 (13, 77) | -0.56 | -2.2% | 26.1 | 0.92 |
| Rule([17](#_ENREF_17)) | 24.6 (12, 89) | -1.0 | -5.8% | 25.3 | 1.0 |

**Supplementary 2 Table E: Bias and Precision of ACR and eAER compared to mAER in patients with mGFR >=60 ml/min/1.73m^2^ (Stage 1 and 2 CKD)**

Median Bias: estimated value (either ACR or eAER) - measured value (mAER).

% Median Bias: ((estimated value (either ACR or eAER) – measured value (mAER)/ measured value (mAER))*100

Precision: Interquartile range (IQR) of median bias.

ACR: Albumin excretion rate calculated from albumin-creatinine ratio.

eAER: Expected albumin excretion rate.

mAER: Measured albumin excretion rate (24-hour urine albumin).

P-value is for comparison between eAER or ACR and mAER.

*Indicates statistically significant result (P<0.007 considered statistically significant with Bonferroni correction for multiple comparisons; See Methods).

mGFR: Measured glomerular filtration rate

**Supplementary 2 Table F: Bias and Precision of ACR and eAER compared to mAER in patients with mGFR 30 to < 60 ml/min/1.73m^2^ (stage 3 CKD)**

| **N= 79** | **Median (IQR) Value [mg/day]** | **Median Bias  [mg/24h]** | **% Median Bias** | **Precision**  **[mg/24h]** | **P Value** |
| --- | --- | --- | --- | --- | --- |
| mAER | 34 (14, 269) | -- | - | -- | -- |
| ACR | 27.4 (11, 121) | -6.4 | -30% | 30.7 | **<0.01*** |
| **eAER by:** |  |  |  |  |  |
| Fotheringham([9](#_ENREF_9)) | 37.1 (14, 155) | -0.9 | -8.1% | 22.2 | 0.43 |
| CKD-EPI([13](#_ENREF_13)) | 36.4 (13, 149) | -1.7 | -5.3% | 25.2 | 0.58 |
| Cockcroft-Gault([14](#_ENREF_14)) | 30.9 (13, 141) | -3.6 | -21.3% | 23.5 | 0.01 |
| Walser([15](#_ENREF_15)) | 34.6 (13, 156) | -2.3 | -11.6% | 27.3 | 0.12 |
| Goldwasser([16](#_ENREF_16)) | 34.2 (14, 162) | -2.7 | -17.1% | 26.6 | 0.07 |
| Rule([17](#_ENREF_17)) | 35.1 (13, 155) | -1.9 | -7.7% | 26.6 | 0.22 |

Median Bias: estimated value (either ACR or eAER) - measured value (mAER).

% Median Bias: ((estimated value (either ACR or eAER) – measured value (mAER)/ measured value (mAER))*100

Precision: Interquartile range (IQR) of median bias.

ACR: Albumin excretion rate calculated from albumin-creatinine ratio.

eAER: Expected albumin excretion rate.

mAER: Measured albumin excretion rate (24-hour urine albumin).

P-value is for comparison between eAER or ACR and mAER.

*Indicates statistically significant result (P<0.007 considered statistically significant with Bonferroni correction for multiple comparisons; See Methods).

mGFR: Measured glomerular filtration rate

**Supplementary 2 Table G: Bias and Precision of ACR and eAER compared to mAER in patients with mGFR < 30 ml/min/1.73m^2^ (Stage 4 and 5 CKD)**

| **N= 18** | **Median (IQR) Value [mg/day]** | **Median Bias  [mg/24h]** | **% Median Bias** | **Precision**  **[mg/24h]** | **P Value** |
| --- | --- | --- | --- | --- | --- |
| mAER | 215 (45, 1409) | -- | - | -- | -- |
| ACR | 144.1 (42, 735) | -12.1 | -6.0% | 260.6 | 0.14 |
| **eAER by:** |  |  |  |  |  |
| Fotheringham([9](#_ENREF_9)) | 206.1 (69, 1280) | 15.5 | 30.9% | 207.8 | 0.39 |
| CKD-EPI([13](#_ENREF_13)) | 213.1 (62, 1043) | 23.8 | 24.4% | 153 | 0.12 |
| Cockcroft-Gault([14](#_ENREF_14)) | 190.2 (51, 916) | -6.2 | -5.7% | 128.3 | 1.0 |
| Walser([15](#_ENREF_15)) | 209.6 (61, 984) | 9.7 | 12.9% | 162.2 | 0.23 |
| Goldwasser([16](#_ENREF_16)) | 215.9 (67, 873) | 8.1 | 10.5% | 155.2 | 0.37 |
| Rule([17](#_ENREF_17)) | 203 (74, 1046) | 17.2 | -7.7% | 147.7 | 0.25 |

Median Bias: estimated value (either ACR or eAER) - measured value (mAER).

% Median Bias: ((estimated value (either ACR or eAER) – measured value (mAER)/ measured value (mAER))*100

Precision: Interquartile range (IQR) of median bias.

ACR: Albumin excretion rate calculated from albumin-creatinine ratio.

eAER: Expected albumin excretion rate.

mAER: Measured albumin excretion rate (24-hour urine albumin).

P-value is for comparison between eAER or ACR and mAER.

No result was statistically significant (P<0.007 considered statistically significant with Bonferroni correction for multiple comparisons; See Methods).

mGFR: Measured glomerular filtration rate

**Supplementary 2 Table H: Accuracy of ACR and eAER compared to mAER in patients with mGFR >= 60 ml/min/1.73m^2^ (Stage 1 and 2 CKD)**

| **N= 84** | **P_15%_** | **P-value^α^** | **P_30%_** | **P-value^β^** | **P_50%_** | **P-value^µ^** |
| --- | --- | --- | --- | --- | --- | --- |
| ACR | 18 (10, 28) | - | 36 (26, 47) | - | 60 (48,70) | - |
| **eAER by:** |  |  |  |  |  |  |
| Fotheringham([9](#_ENREF_9)) | 19 (11, 29) | 0.83 | 42 (31, 53) | 0.38 | 60 (48, 70) | 1 |
| CKD-EPI([13](#_ENREF_13)) | 21 (13, 32) | 0.56 | 42 (31, 53) | 0.34 | 66 (54, 76) | 0.23 |
| Cockcroft-Gault([14](#_ENREF_14)) | 17 (9, 26) | 0.84 | 48 (37, 59) | 0.05 | 64 (53, 75) | 0.29 |
| Walser([15](#_ENREF_15)) | 20 (12, 30) | 0.7 | 42 (31, 53) | 0.38 | 66 (54, 76) | 0.12 |
| Goldwasser([16](#_ENREF_16)) | 29 (19, 40) | 0.1 | 48 (37, 59) | 0.06 | 64 (53, 75) | 0.29 |
| Rule([17](#_ENREF_17)) | 16 (9, 25) | 0.66 | 45 (34, 57) | 0.12 | 64 (53, 75) | 0.32 |

P_1 5%,_ P_30%,_ P_50%:_ Proportion of ACR or eAER within 15%, 30% and 50% of reference standard (measured 24-hour urine albumin) respectively.

^α^P, ^β^P, ^µ^P: P-value for comparison between accuracy of eAER vs accuracy of ACR for P_15%,_  P_30%_ and P_50%_ respectively.

ACR: Albumin excretion rate calculated from albumin-creatinine ratio.

eAER: Expected albumin excretion rate.

No result was statistically significant (P<0.008 considered statistically significant with Bonferroni correction for multiple comparisons; See Methods).

**Supplementary 2 Table I: Accuracy of ACR and eAER compared to mAER in patients with mGFR 30 to <60 ml/min/1.73m^2^ (Stage 3 CKD)**

| **N= 79** | **P_15%_** | **P-value^α^** | **P_30%_** | **P-value^β^** | **P_50%_** | **P-value^µ^** |
| --- | --- | --- | --- | --- | --- | --- |
| ACR | 15 (8, 25) | - | 37 (26, 48) | - | 66 (54, 76) | - |
| **eAER by:** |  |  |  |  |  |  |
| Fotheringham([9](#_ENREF_9)) | 29 (5, 19) | 0.03 | 56 (41, 82) | 0.03 | 71 (60, 81) | 0.29 |
| CKD-EPI([13](#_ENREF_13)) | 27 (17, 38) | 0.83 | 56 (44, 67) | **<0.01*** | 75 (64, 84) | 0.04 |
| Cockcroft-Gault([14](#_ENREF_14)) | 19 (11, 29) | 0.44 | 48 (37, 60) | 0.07 | 73 (62, 83) | 0.08 |
| Walser([15](#_ENREF_15)) | 25 (16, 36) | 0.1 | 56 (44, 67) | **<0.01*** | 73 (62, 83) | 0.11 |
| Goldwasser([16](#_ENREF_16)) | 18 (10, 28) | 0.59 | 42 (31, 53) | 0.45 | 70 (58, 80) | 0.41 |
| Rule([17](#_ENREF_17)) | 33 (21, 44) | 0.35 | 54 (43, 66) | <**0.01*** | 72 (61, 82) | 0.17 |

P_1 5%,_ P_30%,_ P_50%:_ Proportion of ACR or eAER within 15%, 30% and 50% of reference standard (measured 24-hour urine albumin) respectively.

^α^P, ^β^P, ^µ^P: P-value for comparison between accuracy of eAER vs accuracy of ACR for P_15%,_  P_30%_ and P_50%_ respectively.

ACR: Albumin excretion rate calculated from albumin-creatinine ratio.

eAER: Expected albumin excretion rate.

*Indicates statistically significant result (P<0.008 considered statistically significant with Bonferroni correction for multiple comparisons; See Methods).

**Supplementary 2 Table J: Accuracy of ACR and eAER compared to mAER in patients with mGFR < 30 ml/min/1.73m^2^ (Stage 4 and 5 CKD)**

| **N= 18** | **P_15%_** | **P-value^α^** | **P_30%_** | **P-value^β^** | **P_50%_** | **P-value^µ^** |
| --- | --- | --- | --- | --- | --- | --- |
| ACR | 28 (10, 54) | - | 56 (31, 79) | - | 72 (47, 90) | - |
| **eAER by:** |  |  |  |  |  |  |
| Fotheringham([9](#_ENREF_9)) | 22 (6, 48) | 0.71 | 39 (17, 64) | 0.32 | 56 (31, 79) | 0.18 |
| CKD-EPI([13](#_ENREF_13)) | 22 (6, 48) | 0.74 | 44 (22, 69) | 0.53 | 67 (41, 87) | 0.66 |
| Cockcroft-Gault([14](#_ENREF_14)) | 39 (17, 64) | 0.48 | 50 (26, 74) | 0.71 | 72 (47, 90) | 1 |
| Walser([15](#_ENREF_15)) | 33 (13, 59) | 0.71 | 56 (31, 79) | 1 | 67 (41, 87) | 0.56 |
| Goldwasser([16](#_ENREF_16)) | 44 (22, 69) | 0.26 | 61 (36, 83) | 0.66 | 78 (52, 94) | 0.56 |
| Rule([17](#_ENREF_17)) | 33 (13, 59) | 0.71 | 44 (22, 69) | 0.48 | 61 (36, 83) | 0.41 |

P_1 5%,_ P_30%,_ P_50%:_ Proportion of ACR or eAER within 15%, 30% and 50% of reference standard (measured 24-hour urine albumin) respectively.

^α^P, ^β^P, ^µ^P: P-value for comparison between accuracy of eAER vs accuracy of ACR for P_15%,_  P_30%_ and P_50%_ respectively.

ACR: Albumin excretion rate calculated from albumin-creatinine ratio.

eAER: Expected albumin excretion rate.

No result was statistically significant (P<0.008 considered statistically significant with Bonferroni correction for multiple comparisons; See Methods).

**Supplementary 2 Table K:** **Bias and Precision of PCR and ePER compared to mPER with Proteinuria < 1 gram per day**

| **N=162** | **Median (IQR) Value [mg/day]** | **Median Bias  [mg/24h]** | **% Median Bias** | **Precision**  **[mg/24h]** | **P Value** |
| --- | --- | --- | --- | --- | --- |
| mPER | 180 (70, 333) | - |  | - | - |
| PCR | 139 (83, 228) | -32 | -20.7% | 121 | **<0.01*** |
| **ePER by:** |  |  |  |  |  |
| Fotheringham([9](#_ENREF_9)) | 171 (108, 319) | 8 | 4.8% | 112 | 0.21 |
| CKD-EPI([13](#_ENREF_13)) | 174 (111, 345) | 14 | 11.2% | 100 | 0.1 |
| Cockcroft-Gault([14](#_ENREF_14)) | 156 (86, 294) | -12 | -7.4% | 121 | 0.04 |
| Walser([15](#_ENREF_15)) | 173 (100, 324) | 5 | 4.2% | 111 | 0.52 |
| Goldwasser([16](#_ENREF_16)) | 169 (101, 315) | 1 | 0.3% | 104 | 0.9 |
| Rule([17](#_ENREF_17)) | 172 (103, 321) | 8 | 4.7% | 85 | 0.49 |

Median Bias: estimated value (either PCR or ePER) - measured value (mPER).

% Median Bias: ((estimated value (either PCR or ePER) – measured value (mPER)/ measured value (mPER))*100

Precision: Interquartile range (IQR) of median bias.

PCR: Protein excretion rate calculated from protein-creatinine ratio.

ePER: Expected protein excretion rate.

mPER: Measured protein excretion rate (24-hour urine protein).

P-value is for comparison between ePER or PCR and mPER.

*Indicates statistically significant result (P<0.007 considered statistically significant with Bonferroni correction for multiple comparisons; See Methods).

**Supplementary 2 Table L:** **Bias and Precision of PCR and ePER compared to mPER with Proteinuria >1 gram per day**

| **N=19** | **Median (IQR) Value [mg/day]** | **Median Bias  [mg/24h]** | **% Median Bias** | **Precision**  **[mg/24h]** | **P Value** |
| --- | --- | --- | --- | --- | --- |
| mPER | 1800 | - |  | - | - |
| PCR | 1222 (486, 2345) | -559 | -35% | 919 | **<0.01*** |
| **ePER by:** |  |  |  |  |  |
| Fotheringham([9](#_ENREF_9)) | 1590 (8444, 2712) | -104 | -6.3% | 1346 | 0.57 |
| CKD-EPI([13](#_ENREF_13)) | 1636 (1020, 2358) | -153 | -10.3% | 1054 | 0.57 |
| Cockcroft-Gault([14](#_ENREF_14)) | 1551(834, 1962) | -305 | -28.7% | 1124 | 0.09 |
| Walser([15](#_ENREF_15)) | 1669 (915, 2319) | -241 | -21.8% | 1377 | 0.33 |
| Goldwasser([16](#_ENREF_16)) | 1547 (825, 2222) | -325 | -18.7% | 1198 | 0.2 |
| Rule([17](#_ENREF_17)) | 1754 (1065, 2417) | -249 | -15% | 1344 | 0.35 |

Median Bias: estimated value (either PCR or ePER) - measured value (mPER).

% Median Bias: ((estimated value (either PCR or ePER) – measured value (mPER)/ measured value (mPER))*100

Precision: Interquartile range (IQR) of median bias.

PCR: Protein excretion rate calculated from protein-creatinine ratio.

ePER: Expected protein excretion rate.

mPER: Measured protein excretion rate (24-hour urine protein).

P-value is for comparison between ePER or PCR and mPER.

*Indicates statistically significant result (P<0.007 considered statistically significant with Bonferroni correction for multiple comparisons; See Methods).

**Supplementary 2 Table M: Accuracy of PCR and ePER compared to mPER with Proteinuria < 1 gram per day**

| **N= 162** | **P_15%_** | **P-value^α^** | **P_30%_** | **P-value^β^** | **P_50%_** | **P-value^µ^** |
| --- | --- | --- | --- | --- | --- | --- |
| PCR | 21 (15, 28) | - | 44 (36, 52) | - | 49 (41, 57) | - |
| **ePER by:** |  |  |  |  |  |  |
| Fotheringham([9](#_ENREF_9)) | 24 (17, 31) | 0.53 | 45 (37, 53) | 0.81 | 57 (49, 65) | 0.05 |
| CKD-EPI([13](#_ENREF_13)) | 24 (18, 31) | 0.48 | 53 (45, 61) | 0.05 | 60 (52, 68) | 0.02 |
| Cockcroft-Gault([14](#_ENREF_14)) | 25 (18, 32) | 0.4 | 49 (41, 57) | 0.22 | 54 (46, 62) | 0.21 |
| Walser([15](#_ENREF_15)) | 27 (20, 34) | 0.19 | 52 (44, 60) | 0.08 | 59 (51, 67) | 0.02 |
| Goldwasser([16](#_ENREF_16)) | 27 (20, 34) | 0.21 | 46 (38, 54) | 0.56 | 56 (48, 64) | 0.07 |
| Rule([17](#_ENREF_17)) | 28 (21, 35) | 0.1 | 51 (43, 59) | 0.11 | 73 (65, 80) | **< 0.01*** |

P_15%,_ P_30%,_ P_50%:_ Proportion of PCR or ePER within 15%, 30% and 50% of reference standard (measured 24-hour urine protein) respectively.

^α^P, ^β^P, ^µ^P: P-value for comparison between accuracy of ePER vs accuracy of PCR for P_15%,_  P_30%_ and P_50%_ respectively.

PCR: Protein excretion rate calculated from protein-creatinine ratio.

ePER: Expected protein excretion rate.

*Indicates statistically significant result (P<0.008 considered statistically significant with Bonferroni correction for multiple comparisons; See Methods).

**Supplementary 2 Table N: Accuracy of PCR and ePER compared to mPER with Proteinuria > 1 gram per day**

| **N= 19** | **P_15%_** | **P-value^α^** | **P_30%_** | **P-value^β^** | **P_50%_** | **P-value^µ^** |
| --- | --- | --- | --- | --- | --- | --- |
| PCR | 32 (13, 57) | - | 32 (13, 57) | - | 37 (16, 62) | - |
| **ePER by:** |  |  |  |  |  |  |
| Fotheringham([9](#_ENREF_9)) | 21 (6, 46) | 0.34 | 58 (34, 80) | 0.13 | 63 (38, 84) | 0.1 |
| CKD-EPI([13](#_ENREF_13)) | 37 (16, 62) | 0.78 | 58 (34, 80) | 0.17 | 63 (38, 84) | 0.13 |
| Cockcroft-Gault([14](#_ENREF_14)) | 26 (9, 51) | 0.71 | 42 (20, 67) | 0.53 | 53 (29, 76) | 0.26 |
| Walser([15](#_ENREF_15)) | 16 (3, 40) | 0.26 | 53 (29, 76) | 0.21 | 63 (39, 84) | 0.1 |
| Goldwasser([16](#_ENREF_16)) | 21 (6, 46) | 0.41 | 53 (29, 76) | 0.16 | 58 (34, 80) | 0.16 |
| Rule([17](#_ENREF_17)) | 16 (3, 40) | 0.32 | 63 (38, 84) | 0.08 | 74 (49, 91) | 0.04 |

P_15%,_ P_30%,_ P_50%:_ Proportion of PCR or ePER within 15%, 30% and 50% of reference standard (measured 24-hour urine protein) respectively.

^α^P, ^β^P, ^µ^P: P-value for comparison between accuracy of ePER vs accuracy of PCR for P_15%,_  P_30%_ and P_50%_ respectively.

PCR: Protein excretion rate calculated from protein-creatinine ratio.

ePER: Expected protein excretion rate.

No result was statistically significant (P<0.008 considered statistically significant with Bonferroni correction for multiple comparisons; See Methods).

**Supplementary 2 Table O:** **Bias and Precision of PCR and ePER compared to mPER in patients with mGFR >=60 ml/min/1.73m^2^ (Stage 1 and 2 CKD)**

| **N= 84** | **Median (IQR) Value [mg/day]** | **Median Bias  [mg/24h]** | **%Median**  **Bias** | **Precision**  **[mg/24h]** | **P Value** |
| --- | --- | --- | --- | --- | --- |
| mPER | 150 (70, 290) | - | - | - | - |
| PCR | 122 (79, 203) | -23 | -16.9% | 116.2 | **<0.01*** |
| **ePER by:** |  |  |  |  |  |
| Fotheringham([9](#_ENREF_9)) | 147 (100, 270) | 11 | 10.1% | 107 | 0.31 |
| CKD-EPI([13](#_ENREF_13)) | 160 (105, 262) | 14 | 13.4% | 111.5 | 0.28 |
| Cockcroft-Gault([14](#_ENREF_14)) | 141 (86, 239) | -7 | -6.1% | 98.6 | 0.33 |
| Walser([15](#_ENREF_15)) | 160 (100, 268) | 12 | 6.7% | 105.7 | 0.36 |
| Goldwasser([16](#_ENREF_16)) | 162 (99, 262) | 6 | 2.4% | 85.9 | 0.47 |
| Rule([17](#_ENREF_17)) | 148, 97, 253) | 9 | 6.1% | 108.8 | 0.66 |

Median Bias: estimated value (either PCR or ePER) - measured value (mPER).

% Median Bias: ((estimated value (either PCR or ePER) – measured value (mPER)/ measured value (mPER))*100

Precision: Interquartile range (IQR) of median bias.

PCR: Protein excretion rate calculated from protein-creatinine ratio.

ePER: Expected protein excretion rate.

mPER: Measured protein excretion rate (24-hour urine protein).

P-value is for comparison between ePER or PCR and mPER.

*Indicates statistically significant result (P<0.007 considered statistically significant with Bonferroni correction for multiple comparisons; See Methods).

**Supplementary 2 Table P:** **Bias and Precision of PCR and ePER compared to mPER in patients with mGFR 30 to <60 ml/min/1.73m^2^ (Stage 3 CKD)**

| **N= 79** | **Median (IQR) Value [mg/day]** | **Median Bias  [mg/24h]** | **%Median**  **Bias** | **Precision**  **[mg/24h]** | **P Value** |
| --- | --- | --- | --- | --- | --- |
| mPER | 220 (90, 520) | - | - | - | - |
| PCR | 155 (86, 343) | -60 | -27.3% | 172.1 | **<0.01*** |
| **ePER by:** |  |  |  |  |  |
| Fotheringham([9](#_ENREF_9)) | 195 (116, 506) | -11 | -5.6% | 134.3 | 0.63 |
| CKD-EPI([13](#_ENREF_13)) | 202 (111, 468) | -2 | -1.77% | 143.4 | 0.65 |
| Cockcroft-Gault([14](#_ENREF_14)) | 185 (83, 425) | -24 | -18.7 | 165.4 | **<0.01*** |
| Walser([15](#_ENREF_15)) | 213 (99, 462) | -14 | -6.4% | 153 | 0.19 |
| Goldwasser([16](#_ENREF_16)) | 207 (101, 445) | -21 | -9.9% | 158.8 | 0.07 |
| Rule([17](#_ENREF_17)) | 190 (104, 449) | -6 | -5.0% | 119.5 | 0.22 |

Median Bias: estimated value (either PCR or ePER) - measured value (mPER).

% Median Bias: ((estimated value (either PCR or ePER) – measured value (mPER)/ measured value (mPER))*100

Precision: Interquartile range (IQR) of median bias.

PCR: Protein excretion rate calculated from protein-creatinine ratio.

ePER: Expected protein excretion rate.

mPER: Measured protein excretion rate (24-hour urine protein).

P-value is for comparison between ePER or PCR and mPER.

*Indicates statistically significant result (P<0.007 considered statistically significant with Bonferroni correction for multiple comparisons; See Methods).

**Supplementary 2 Table Q:** **Bias and Precision of PCR and ePER compared to mPER in patients with mGFR < 30 ml/min/1.73m^2^ (Stage 4 and 5 CKD)**

| **N= 18** | **Median (IQR) Value [mg/day]** | **Median Bias  [mg/24h]** | **%Median**  **Bias** | **Precision**  **[mg/24h]** | **P Value** |
| --- | --- | --- | --- | --- | --- |
| mPER | 450 (260, 1650) | - | - | - | - |
| PCR | 526 (228, 1246) | -53 | -9.7% | 263.5 | 0.23 |
| **ePER by:** |  |  |  |  |  |
| Fotheringham([9](#_ENREF_9)) | 677 (319, 2171) | 113 | 32.1% | 374.5 | 0.2 |
| CKD-EPI([13](#_ENREF_13)) | 612 (345, 1769) | 80 | 16.2% | 240.9 | 0.14 |
| Cockcroft-Gault([14](#_ENREF_14)) | 491 (296, 1553) | -6 | -5.8% | 269.1 | 0.9 |
| Walser([15](#_ENREF_15)) | 545 (342, 1672) | 58 | 12.4% | 223.6 | 0.33 |
| Goldwasser([16](#_ENREF_16)) | 497 (315, 1488) | 47 | 10.5% | 282.9 | 0.42 |
| Rule([17](#_ENREF_17)) | 642 (301, 1773) | 73 | 15.2% | 277.7 | 0.3 |

Median Bias: estimated value (either PCR or ePER) - measured value (mPER).

% Median Bias: ((estimated value (either PCR or ePER) – measured value (mPER)/ measured value (mPER))*100

Precision: Interquartile range (IQR) of median bias.

PCR: Protein excretion rate calculated from protein-creatinine ratio.

ePER: Expected protein excretion rate.

mPER: Measured protein excretion rate (24-hour urine protein).

P-value is for comparison between ePER or PCR and mPER.

No result was statistically significant (P<0.007 considered statistically significant with Bonferroni correction for multiple comparisons; See Methods).

**Supplementary 2 Table R: Accuracy of PCR and ePER compared to mPER in patients with mGFR >=60 ml/min/1.73m^2^ (Stage 1 and 2 CKD)**

| **N= 84** | **P_15%_** | **P-value^α^** | **P_30%_** | **P-value^β^** | **P_50%_** | **P-value^µ^** |
| --- | --- | --- | --- | --- | --- | --- |
| PCR | 25 (16, 36) | - | 44 (33, 55) | - | 48 (38, 60) | - |
| **ePER by:** |  |  |  |  |  |  |
| Fotheringham([9](#_ENREF_9)) | 26 (17, 37) | 0.85 | 44 (33, 55) | 1 | 56 (45, 67) | 0.22 |
| CKD-EPI([13](#_ENREF_13)) | 20 (12, 30) | 0.35 | 50 (39, 61) | 0.35 | 57 (46, 68) | 0.18 |
| Cockcroft-Gault([14](#_ENREF_14)) | 26 (17, 37) | 0.85 | 46 (36, 58) | 0.68 | 52 (41, 63) | 0.51 |
| Walser([15](#_ENREF_15)) | 26 (17, 37) | 0.84 | 50 (39, 61) | 0.34 | 57 (46, 68) | 0.18 |
| Goldwasser([16](#_ENREF_16)) | 29 (19, 40) | 0.58 | 50 (39, 61) | 0.32 | 57 (46, 68) | 0.14 |
| Rule([17](#_ENREF_17)) | 25 (16, 36) | 1 | 51 (40, 62) | 0.26 | 68 (57, 78) | <**0.01*** |

P_15%,_ P_30%,_ P_50%:_ Proportion of PCR or ePER within 15%, 30% and 50% of reference standard (measured 24-hour urine protein) respectively.

^α^P, ^β^P, ^µ^P: P-value for comparison between accuracy of ePER vs accuracy of PCR for P_15%,_  P_30%_ and P_50%_ respectively.

PCR: Protein excretion rate calculated from protein-creatinine ratio.

ePER: Expected protein excretion rate.

*Indicates statistically significant result (P<0.008 considered statistically significant with Bonferroni correction for multiple comparisons; See Methods).

**Supplementary 2 Table S: Accuracy of PCR and ePER compared to mPER in patients with mGFR 30 to <60 ml/min/1.73m^2^ (Stage 3 CKD)**

| **N= 79** | **P_15%_** | **P-value^α^** | **P_30%_** | **P-value^β^** | **P_50%_** | **P-value^µ^** |
| --- | --- | --- | --- | --- | --- | --- |
| PCR | 17 (9, 27) | - | 39 (28, 51) | - | 44 (33, 56) | - |
| **ePER by:** |  |  |  |  |  |  |
| Fotheringham([9](#_ENREF_9)) | 22 (13, 32) | 0.29 | 51 (39, 62) | 0.13 | 62 (50, 73) | **<0.01*** |
| CKD-EPI([13](#_ENREF_13)) | 28 (18, 39) | 0.08 | 57 (45, 68) | 0.01 | 62 (50, 73) | 0.01 |
| Cockcroft-Gault([14](#_ENREF_14)) | 23 (14, 34) | 0.28 | 48 (37, 60) | 0.13 | 53 (42, 65) | 0.11 |
| Walser([15](#_ENREF_15)) | 24 (15, 35) | 0.2 | 53 (42, 65) | 0.05 | 61 (49, 72) | 0.01 |
| Goldwasser([16](#_ENREF_16)) | 20 (12, 31) | 0.51 | 39 (28, 51) | 1 | 53 (42, 65) | 0.13 |
| Rule([17](#_ENREF_17)) | 28 (18, 39) | 0.06 | 54 (43, 66) | 0.03 | 80 (69, 88) | **<0.01*** |

P_15%,_ P_30%,_ P_50%:_ Proportion of PCR or ePER within 15%, 30% and 50% of reference standard (measured 24-hour urine protein) respectively.

^α^P, ^β^P, ^µ^P: P-value for comparison between accuracy of ePER vs accuracy of PCR for P_15%,_  P_30%_ and P_50%_ respectively.

PCR: Protein excretion rate calculated from protein-creatinine ratio.

ePER: Expected protein excretion rate.

*Indicates statistically significant result (P<0.008 considered statistically significant with Bonferroni correction for multiple comparisons; See Methods).

**Supplementary 2 Table T: Accuracy of PCR and ePER compared to mPER in patients with mGFR < 30 ml/min/1.73m^2^ (Stage 4 and 5 CKD)**

| **N= 18** | **P_15%_** | **P-value^α^** | **P_30%_** | **P-value^β^** | **P_50%_** | **P-value^µ^** |
| --- | --- | --- | --- | --- | --- | --- |
| PCR | 33 (13, 59) | - | 50 (26, 74) | - | 56 (31, 79) | - |
| **ePER by:** |  |  |  |  |  |  |
| Fotheringham([9](#_ENREF_9)) | 17 (4, 41) | 0.26 | 39 (17, 64) | 0.53 | 50 (26, 74) | 0.74 |
| CKD-EPI([13](#_ENREF_13)) | 39 (17, 64) | 0.76 | 56 (31, 79) | 0.76 | 67 (41, 87) | 0.53 |
| Cockcroft-Gault([14](#_ENREF_14)) | 28 (10, 54) | 0.74 | 56 (31, 79) | 0.71 | 61 (36, 83) | 0.71 |
| Walser([15](#_ENREF_15)) | 28 (10, 54) | 0.71 | 56 (31, 79) | 0.71 | 67 (41, 87) | 0.48 |
| Goldwasser([16](#_ENREF_16)) | 39 (17, 64) | 0.71 | 67 (41, 87) | 0.26 | 67 (41, 87) | 0.48 |
| Rule([17](#_ENREF_17)) | 28 (10, 54) | 0.7 | 50 (26, 74) | 1 | 67 (41, 87) | 0.56 |

P_15%,_ P_30%,_ P_50%:_ Proportion of PCR or ePER within 15%, 30% and 50% of reference standard (measured 24-hour urine protein) respectively.

^α^P, ^β^P, ^µ^P: P-value for comparison between accuracy of ePER vs accuracy of PCR for P_15%,_  P_30%_ and P_50%_ respectively.

PCR: Protein excretion rate calculated from protein-creatinine ratio.

ePER: Expected protein excretion rate.

No result was statistically significant (P<0.008 considered statistically significant with Bonferroni correction for multiple comparisons; See Methods).
